# Using real‐world examples of the COVID‐19 pandemic to increase student confidence in their scientific literacy skills

**DOI:** 10.1002/bmb.21474

**Published:** 2020-11-02

**Authors:** Ashlyn E. Anderson, Louis B. Justement, Heather A. Bruns

**Affiliations:** ^1^ Department of Cell, Developmental and Integrative Biology University of Alabama at Birmingham Birmingham Alabama USA; ^2^ Department of Microbiology University of Alabama at Birmingham Birmingham Alabama USA

**Keywords:** active learning, coronavirus, COVID‐19, immunology, immunology education, real‐world relevance, scientific literacy, undergraduate STEM education

## Abstract

Over the last few decades, there has been a shift in the classroom from lecture‐based to active learning settings with the argument that students retain more information when they are involved in the learning process. This correlation is even stronger when the active learning setting incorporates a real‐world or personal connection. Using active learning activities that develop students' ability to comprehend primary scientific literature is particularly important in the field of immunology, due to the rapid expansion of information in the field, which has been further accelerated due to the COVID‐19 pandemic. By nature, immunology is interdisciplinary, requiring an integrated knowledge of concepts from several scientific disciplines to understand complex immune processes. Engaging undergraduate students through the use of primary literature can improve scientific literacy, develop critical thinking, and enhance understanding of complex topics. To explore this, we utilized a group learning activity in an introductory immunology course that incorporated both a coronavirus‐related review and COVID‐19 clinical research article. We found that this learning activity significantly enhanced student confidence in key scientific literacy skills: reading scientific literature, clearly explaining relevant points, and describing conclusions generated from the data. Moreover, all students reported that they enjoyed the activity and that it helped them understand more about the current COVID‐19 pandemic in the context of the immune response.

## 1 INTRODUCTION

Immunology is, by nature, interdisciplinary and requires an understanding of concepts across an array of scientific disciplines in order to fully grasp the function of individual components as well as the immune system as a whole. Furthermore, discussion focused on immunology‐based experimental techniques, such as flow cytometry or enzyme‐linked immunosorbant assay (ELISA), requires students to apply their knowledge from several scientific disciplines in order to fully understand the experimental process. The comprehension of complex immunologic concepts and experimental techniques can be enhanced through reading and comprehending scientific literature.

Indeed, a critical component of science education is fostering the development of information literacy.^1^, ^2^ Scientific writing requires familiarity with academic styles that are defined in a variety of ways, depending on the scientific discipline.^3^ The ability to fully comprehend scientific literature is challenging even by students able to read accurately and fluently.^4^, ^5^ Moreover, comprehension of the primary literature depends on students having at least a rudimentary understanding of experimental techniques, and how to analyze the data obtained. The ability to read primary scientific literature can help fill the information gap between material covered in textbooks and newly emerging data and evidence for revision of prior understanding.^6^ This is particularly important in the field of immunology, in which information is rapidly expanding and a vast number of scientific articles detailing new findings are published daily.

Information literacy can be demonstrated in a variety of ways, including an understanding of appropriate databases used to search for information, as well as the ability to construct an effective literature search, to validate appropriate sources, to extract relevant information, and to synthesize and communicate key concepts.^7^, ^8^ Identifying and implementing activities that develop these skills can be difficult and often viewed by students as less enjoyable than educational activities focused on subject‐specific knowledge. The use of active learning pedagogies can aid in contextual understanding and student engagement and are often associated with enhanced retention of information, application of knowledge, critical thinking skills, and communication skills.^9^, ^10^, ^11^, ^12^, ^13^ In many disciplines, including science, technology, engineering, and mathematics (STEM) courses, there has been a shift to active learning, often in the form of team‐based learning (TBL), case studies or poll questions, and problem‐solving exercises. Studies comparing active learning to lecture based learning in STEM courses have provided evidence to suggest that active learning positively correlates with enhanced academic achievement.^9^, ^14^ Furthermore, the use of case studies is not only favorably viewed by students but can also enhance critical thinking skills and help students apply what they learn in the classroom to relevant, real‐life situations.^10^, ^11^ Furthermore, learning gains associated with case studies are significantly higher compared to class discussions and textbook readings.^12^ Importantly, real‐world examples aid in learning. Thus, medical‐based curricula strongly encourage the introduction of real‐world clinical context into lessons when teaching about immunological concepts.^15^, ^16^

The recent outbreak of COVID‐19 provides an opportunity to reinforce fundamental topics commonly presented in an introductory immunology course (or course that includes a unit on the immune system) and to expand student understanding through discussion of a current, relevant event and how this relates to the immune response. The rapidly evolving nature of information dissemination about COVID‐19 requires that students read scientific articles to acquire knowledge about the subject, and thus, additionally provides the opportunity to develop students' information literacy and communication skills. The goal of this study was to assess students' perception of the effectiveness of incorporating the real‐world relevance of the COVID‐19 pandemic into an information literacy learning activity focused on developing scientific literacy skills and enhancing understanding of fundamental concepts in immunology. Our hypothesis was that learning about an ongoing, real‐world pandemic that has direct relevance to the immune system will positively influence students' perceived understanding of complex immunological concepts and increase student confidence in reading and comprehending scientific literature.

## 2 METHODS

Students participating in this learning activity were predominantly sophomores majoring in immunology. The University of Alabama at Birmingham offers a unique undergraduate major in immunology, the Undergraduate Immunology Program (UIP), through a partnership between the Department of Microbiology in the School of Medicine and the Department of Biology in the College of Arts and Sciences.^17^ A majority of students in this major are focused on careers in the health professions and will seek acceptance into professional or graduate schools in the biomedical sciences. All students participating in this learning activity were enrolled in MIC 275 (Introduction to the Immune System), the first of five core courses that teach foundational concepts in immunology in the UIP.

The purpose of the assignment, titled Coronavirus Article Analysis, was to reinforce students' understanding of immunology concepts in an engaging context while developing their ability to read scientific articles, extract relevant information, analyze data figures, and convey information to peers. The learning objectives for the assignment were:

1. Extract appropriate information from a scientific review article to describe the innate and adaptive immune responses to coronaviruses.
2. Understand how to analyze a figure from a scientific paper by identifying the hypothesis to be tested, experimental design/approach, and interpret the data presented to understand the conclusion of the figure.
3. Understand how each experiment in a scientific paper contributes to the broader observations/conclusions of the paper.
4. Identify and communicate relevant scientific information with peers.

### 2.1 Procedure

The activity took place over 2 days for 1 h and 15 min each day, and teams of students worked together in breakout groups via Zoom. Three instructors participated in the activity, and a facilitator guide was provided (Appendix S1). All information and components of the activity that are described below are included in the facilitator guide. Students were given the assignment in its entirety prior to class and were asked to read it in its entirety to know what to expect during the class.

### 2.2 Part 1 of the activity

Prior to the first day, students were assigned a review article titled “COVID‐19: Immune responses in COVID‐19 and potential vaccines: Lessons learned from SARS and MERS epidemic” to read .^18^ Immediately after entering the Zoom chat, students took a five‐question quiz covering main concepts from the article. Upon completion, students were randomly assigned to a group to work in Zoom breakout rooms. There were four groups consisting of up to four students each. Two groups were asked to discuss the information in the review article that related to the innate immune response to coronaviruses, while the other two groups were tasked with discussing the adaptive immune response to coronaviruses. Students were given a list of questions to consider with their group as they discussed the article. These questions were specific to components of the innate or adaptive immune response and are listed in the facilitator guide (Appendix S1). Students were instructed that as a group, they needed to create a short PowerPoint presentation of no more than four slides and be prepared to share their understanding of the immune response against coronaviruses with the other groups. During the group work session, the instructors joined different breakout rooms as facilitators to assist groups if they had any questions. After 30 min of group work, students emailed the group PowerPoint files to the instructor, left their breakout room, and returned to the main Zoom chat. Each of the two innate immune response groups spent 5 min giving their presentation, followed by 5 min of discussion by the whole class. The two remaining groups then presented their information on the adaptive response followed by a whole class discussion. To ensure individual accountability, an additional component to the assignment was that each student had to submit a written paragraph summarizing the group's findings by the end of the day.

### 2.3 Part 2 of the activity

Prior to the second day, students were assigned to read the primary journal article: “Distinct Immune Response in Two MERS‐CoV‐Infected Patients: Can We Go from Bench to Bedside?”.^19^ The class meeting was again held via a Zoom chat and then upon entering the chat, students took a five‐question quiz covering the main points of the paper. In contrast to the first session, which required students to read a review article, the second session required students to read a primary journal article and the focus of the activity was for students to analyze a figure from the paper. Immediately following the quiz, the instructor went over the key differences between a review and a primary journal article and the purpose of both written formats. Students were again randomly assigned to a group to work in Zoom breakout rooms. Each group was assigned one figure from the paper and instructed to create a short PowerPoint presentation that included the following:

1. State the figure number that you are analyzing.
2. Create a simple title for your figure that describes what is being done.
3. State the hypothesis that is being tested or the question being answered by this experiment. What is it that the researchers want to know by doing this experiment?
4. Each figure uses at least one experimental technique. State this technique. Find a picture to explain what this technique is and what type of information (data) is obtained. In your explanation of this experimental technique, consider the following questions:
  - What is being measured and how?
  - What is being compared? What are the control(s) and the experimental variable(s)?
5. When looking at the data presented in the figure, answer the following question.
  - What are the conclusions that can be drawn?
6. What is another experimental technique that the authors could have employed to make the same conclusion?

Instructors visited the breakout groups to address any concerns or questions. After 30 min of group work, students emailed the group PowerPoint files to the instructor, left their breakout group, and returned to the main Zoom chat. Each group was given 5 min to give their presentation followed by a few minutes of whole class discussion. Similar to the first day, an additional component to the assignment was that each student had to submit a written paragraph summarizing the group's findings by the end of the day.

#### 2.3.1 *Informed consent for research involving human subjects*

Data from preevaluation and postevaluation responses were collected anonymously. In addition, the information provided in this report is from a learning activity in one course and not part of an ongoing study. However, the Undergraduate Immunology Program has approval by the University of Alabama at Birmingham Institutional Review Board to assess curricular activities within program courses (IRB‐300003280).

#### 2.3.2 *Data collection and analysis*

**FIGURE 1:**
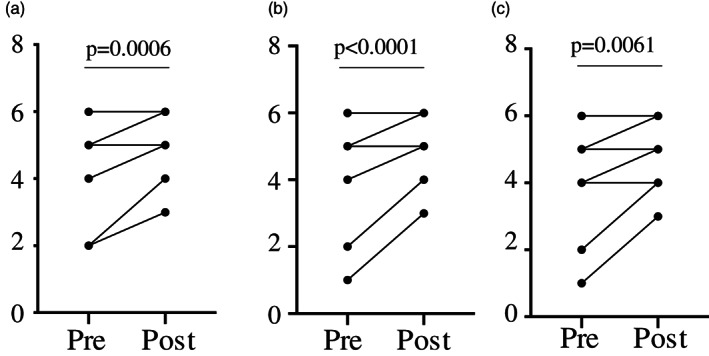
Graphical representation of self‐assessment evaluation questions. (a) **“**I can read scientific articles and identify the important or relevant points.” (b) “I can read scientific articles and clearly explain the main points to others.” (c) “I can read scientific articles and describe the conclusions made from the data presented in figures”. Some student answers overlap (*n* = 13); data are normally distrusted; a parametric paired *t* test was performed

Thirteen students in MIC 275 participated in the learning activity. At the conclusion of the activity, students were given an anonymous link to a single survey through Qualtrics and asked to rate their perception of their abilities described in the statements prior to and following the learning activity (Table 1 and Figure 1). In the same survey, students were also asked to rate the effectiveness of the activity in helping them better understand immune processes, the immune response to coronaviruses, and their overall enjoyment of the learning activity. The Likert scale survey asked them to provide their assessment on a scale of 1–7, where 1 = neither agree nor disagree, 2 = strongly disagree, 3 = disagree, 4 = somewhat disagree, 5 = somewhat agree, 6 = agree, 7 = strongly agree. All 13 students completed the survey. Statistical analyses of the responses to the pre/post self‐assessment questions were performed using Prism 8 (Graph Pad). A parametric paired *t* test was performed following the D'Agostino and Pearson test for normal distribution.

**TABLE 1:** Self‐assessment evaluation questions

		Pre		Post		
		Mean	SD	Mean	SD	*p* Value
A	I can read scientific articles and identify the important or relevant points.	5.31	1.14	6.08	0.83	0.0006
B	I can read scientific articles and clearly explain the main points to others.	5.08	1.27	6.08	0.83	<0.0001
C	I can read scientific articles and describe the conclusions made from the data presented in figures.	5.31	1.38	6.00	0.88	0.0061

*Note*: Mean, SD, and *p* value between preactivity and postactivity evaluation questions. Data are normally distributed; a parametric paired *t* test was performed.

## 3 RESULTS

Overall, students reported significant increases in confidence regarding their abilities to read scientific articles, extract relevant information, explain main concepts, and draw appropriate conclusions from data figures (Table 1). All three self‐assessment statements demonstrated increases in preactivity to postactivity results that were statistically significant. The shift from lower values to higher values demonstrated an increase in perceived ability by the students (Figure 1).

**FIGURE 2:**
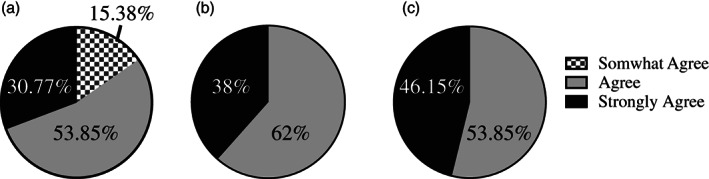
Students perceptions of the COVID‐19 pandemic learning activity. (a) “This activity helped me better understand immunologic processes.” (b) “This activity helped me understand the immune response to coronaviruses.” (c) “Overall I enjoyed this activity”. No student chose the responses neither agree nor disagree, strongly disagree or disagree

Furthermore, all students responded in the affirmative that the learning activity improved their understanding of immunologic processes and the immune response to coronaviruses (Figure 2(ab)). Importantly, when asked if students enjoyed this activity, all students reported a rating of “agree” or “strongly agree” (Figure 2(c)).

## 4 DISCUSSION

The goal of this educational exercise was to develop scientific literacy skills while reinforcing immunology concepts previously covered in the course, and increase student enjoyment in the learning process through the use of a real‐world, current topic. We created a group learning activity focused on the immune response to coronaviruses that required students to read and discuss both review and primary scientific articles. As expected, students enjoyed this learning activity and felt that it increased their understanding of immune processes, particularly concepts specific to immune responses to coronaviruses (Figure 2). This finding supports the hypothesis that the use of real‐world examples enhances students' perceived understanding of immunologic concepts. Furthermore, the results from the self‐assessment pre/post statements (Table 1 and Figure 1) demonstrate the ability of one class activity to improve student confidence in reading scientific articles, clearly explaining the main points and describing the conclusions generated from the data.

Many of the students in this course are pursuing science‐based careers. Reading and understanding scientific literature is an essential skill in many of these careers. It is estimated that scientists spend 23% of their work time reading.^20^ Many scientific papers, including the ones used in this study, have specialized vocabulary and grammatical structure that make it difficult for the reader to understand if one is not familiar with the field. This can cause novice scientists to shy away from reading the literature.^21^, ^22^, ^23^ Hubbard et al. investigated which sections of scientific literature scientists focus on depending on their experience in the field, and found that undergraduates find methods and results sections the most difficult to understand.^23^ In addition, undergraduate students are more likely to rely on conclusions the author makes instead of drawing conclusions for themselves.^24^ For these reasons, this study was designed to have students focus on just one figure of a scientific paper, disassemble each panel, provide a simplified title for the figure, and draw their own conclusions from the data. Although students had read the paper in its entirety before the class, the process of analyzing an individual figure in a group setting provided an opportunity for students to draw their own conclusions from their own interpretation of the data.

Creating learning activities that develop competencies involving analytical reasoning, critical thinking, and communication can be challenging. Further complicating their development is the often negative attitude of the students, who typically view development of skills contributing to these competencies as more difficult and time‐consuming than educational pursuits aimed at learning discipline‐specific content.^25^ However, increased student interest in a topic can often overcome a majority of negative attitudes, particularly when the topics have perceived direct relevance to the student.^16^ In addition to the survey results demonstrating that students enjoyed this learning activity (Figure 2(c)), we observed that students were actively engaged in discussions with their groups, and created a quality work product. It should be noted that this group activity had to take place via Zoom, which can diminish student engagement for a variety of reasons, including technical challenges, lack of familiarity with the online learning interface and difficulties in effectively promoting interpersonal interactions (personal observations). However, despite being in an online format, students put forth a great deal of effort in their breakout groups and during class discussions. Together, this information highlights the importance of creating learning pedagogies, particularly those aimed at developing critical transferrable skills that are perceived negatively by students, that are centered on real world, relevant experiences in order to increase student enjoyment of and engagement in educational activities.

Although the results of the preanalysis/postanalysis demonstrated an increase in student confidence in key information literacy and reasoning skills, we cannot draw any conclusions as to whether increased confidence translated into better performance. A limitation to this study was that we did not directly provide students an opportunity to exhibit mastery of the skills gained from this exercise. Although a majority of students participated in the discussion and took turns explaining concepts, not all students contributed to the discussion. This prohibited an individual assessment of each student's understanding. Inclusion of questions pertaining to figure analysis on an exam, evaluation of student participation in the discussion, or required written summary have been discussed previously as effective means of assessing learning from the use of primary literature as a pedagogical tool.^6^ Thus, implementation of one or more of these assessments could provide a method for demonstrating student learning gains in future implementation of this activity. In addition, we were unable to assess if this learning activity directly improved students' understanding of complex immunology concepts. This can likely be assessed in the future by the inclusion of specific exam questions that target the topics covered in the scientific articles.

Nonetheless, upon self‐evaluation, students reported that they felt this exercise enhanced their understanding of immunology concepts and their ability to read and understand scientific literature, demonstrating an increase in confidence among the students in these areas. Studies on science students in secondary school demonstrate a correlation between self‐confidence and academic achievement.^3^ Furthermore, confidence is a key trait of success in a variety of disciplines and careers, and studies suggest that students who are confident and have realistic expectations exhibit elevated academic performance.^26^, ^27^, ^28^ This exercise not only provided an opportunity to explore immunology concepts in a real‐world context, but also an opportunity to build student confidence in reading and comprehending scientific literature.

This report provides an in‐depth description of an immunology‐focused, COVID‐19‐related learning activity that can be adapted for use in a variety of science courses. The findings in this report support prior published articles demonstrating the positive influence of the inclusion of learning activities with real‐world relevance on student enjoyment and perceived confidence in scientific literacy competencies and provide the basis for future investigations into the effect of incorporating real‐world issues, particularly the COVID‐19 pandemic, into educational activities on a variety of learning outcomes.

## CONFLICT OF INTEREST

The authors declare no potential conflict of interest.

## Supporting information


**APPENDIX** **S1**. Coronavirus article analysis—facilitator guide
