# Supplementary material for: Using real‐world examples of the COVID‐19 pandemic to increase student confidence in their scientific literacy skills
Source: Biochem Mol Biol Educ. 2020 Nov 2;48(6):678–84. doi: 10.1002/bmb.21474 (PMC7756788; doi:10.1002/bmb.21474)
Supplement: Supplementary file 1 — APPENDIX S1. Coronavirus article analysis—facilitator guide [file BMB-48-678-s001.docx]

**Coronavirus Article Analysis – Facilitator Guide**

Introduction

The purpose of this group learning activity is to develop the information literacy, scientific reasoning, teamwork, and communication skills of students and reinforce their understanding of the components of the immune system and their function through analysis and discussion of a review and primary research article on coronaviruses and the COVID-19 pandemic.

Learning Objectives

After completion of this TBL, students will be able to:

1. Extract appropriate information from a scientific review article to describe the innate and adaptive immune responses to coronaviruses.
2. Understand how to analyze a figure from a scientific paper by identifying the hypothesis, experimental design and conclusion of the figure.
3. Understand how each experiment in a scientific paper contributes to the broader observations/conclusions of the paper.
4. Identify and communicate relevant scientific information with peers.

Materials

1. Tuesday: <http://apjai-journal.org/wp-content/uploads/2020/02/Covid_AP-200220-0772.pdf>
2. Thursday: <https://www.ncbi.nlm.nih.gov/pmc/articles/PMC3925152/>

Evaluation (30 points total)

1. Quiz on information from Prompetchara et al – 5 points
2. Group participation for the Prompetchara et al paper discussion – 5 points
3. Individual summary of the Prompetchara et al paper – 5 points
4. Quiz on information from Faurer et al – 5 points
5. Group participation for the Faurer et al figure analysis – 5 points
6. Individual summary of the Faurer et al figure analysis – 5 points

FACILITATOR GUIDE

Activity Time Outline (2.5 hrs total – 1.25h/day)

Note all sessions are now online via Zoom

1. **Prior to class on Day 1:**
   1. Students will have read “Immune responses in COVID-19 and potential vaccines: Lessons learned from SARS and MERS epidemic” (Prompetchara et al)
   2. Students will be prepared to take a short quiz to test recall of information from the paper prior to the start of class.
2. **During class on Day 1:**
   1. Students will first take a quiz on the information in Prompetchara et al review (10 minutes).
   2. Students will be divided into 4 groups. Using the Immune Response Discussion handout (below) as a guide, two of the groups will discuss the innate immune response to MERS-CoV and SARS-CoV discussed in the paper and two of the groups will discuss the adaptive immune response to MERS-CoV and SARS-CoV discussed in the paper. The instructors will be monitoring groups to assess that everyone is participating. Groups will create a short PowerPoint presentation (no more than 4 slides – text and/or pictures can be included) about their topic. (40 minutes)

Reminder: appropriate group participation (answering questions, asking questions and engaging in discussion with the group) is worth 5 points.

Consider the questions:

- - - 1. What are the innate immune responses to viruses and do coronaviruses alter those responses?
      2. Is there a role for antibodies in fighting these viruses? CD4 T cells? CD8 T cells?
  1. Each group will send the instructor their PowerPoint presentation at the end of the discussion.
  2. The instructor will share his/her screen and allow one spokesperson from each group to present their work followed by a class discussion (30 minutes).
  3. Each student will submit (individually) to Canvas a short write up (no more than 150 words) summarizing their group’s presentation. This will be due by the end of day 1.

1. **Prior to class on Day 2:**
   1. Students will have read “Distinct Immune Response in Two MERS-CoV-Infected Patients: Can We Go from Bench to Bedside?” (Faure et al)
   2. Students will be prepared to take a short quiz over the paper prior to the start of class.
2. **During class on Day 2:**
   1. Students will again work in groups to analyze figures from the Faure et al paper. Each group will be assigned one of the following figures: Figure 1A+1G, Figure 2A+2B, Figure 3 or Figure 4. They will answer the questions provided in the Figure Analysis handout (below) and create a PowerPoint. Instructors will be monitoring groups to assess participation.

Reminder: appropriate group participation (answering questions, asking questions and engaging in discussion with the group) is worth 5 points.

- 1. Each group will send the instructor their PowerPoint presentation.
  2. The instructor will share his/her screen and allow one spokesperson from each group to explain the figure followed by a class discussion (30 minutes).
  3. Each student will upload their individual figure analysis to Canvas. This will be due by the end of the day Thursday.

Prompetchara et al Quiz

1. SARS-CoV-2 is thought to originate from a market in Wuhan, China, but there have been reports of people who have the disease that did not have direct contact with the market.

*A. True

B. False

2. Between SARS-CoV, MERS-CoV and SARS-CoV-2, the highest fatality rate (% death/total cases) is with SARS-CoV-2.

A. True

*B. False

3. As seen with SARS-CoV, a possible downfall of the innate immune system during SARS-CoV-2 is diminished type I IFN response.

*A. True

B. False

4. SARS-CoV and SARS-CoV-2 are 100% identical.

A. True

*B. False

5. As expected with a viral disease, there were more SARS-CoV-2 specific CD4 T cells compared to SARS-CoV-2 specific CD8 T cells in infected patients.

A. True

*B. False

Faurer et al. Quiz

1. How many patients did the paper investigate?

A. One

*B. Two

C. One hundred

D. Two thousand and twenty-seven

2. The paper is focused on the late adaptive immune response.

A. True

*B. False

3. The authors of the paper used mouse models to mimic COVID19.

A. True

*B. False

4. Two of the methods described in this paper are qRT-PCR and ELISA.

*A. True

B. False

5. Similar to the paper on Tuesday, Faure et al suggests that IFN(alpha) is a promising pathway to target for a therapy.

*A. True

B. False

Immune Response Discussion (Prompetchara et al)

Innate immune system:

1. Would you expect higher neutrophils and lower lymphocytes for this type of a disease?
2. What innate cell would you expect to be higher during coronavirus? Why?
3. IL-6 and C-reactive protein are higher in severely ill patients. What are these and what cells make them?
4. What is the importance of ACE2?
5. Based on what you know about how viruses infect the immune system, hypothesize what happens when a T cell or macrophage is infected with a coronavirus. Hypothesize how this virus evades the immune system (hint: what could it downregulate?)
6. Explain/ draw out the TLR/RIG-I/PAMP pathway that is hypothesized to be important in the response against coronaviruses.
7. What is the importance of type I IFN and why is it important in the immune response to coronaviruses? What happens if type I IFN response is too low/ too high?

Adaptive immune system:

1. What cell would you expect in the adaptive immune system to play a major role following infection with coronaviruses and why?
2. What is the role of CD4 and CD8 T cells during this disease response?
3. Why are antibodies important for providing protection against coronaviruses?
4. What does seroconversion mean and why is it important?
5. How does the virus evade the adaptive immune response?

Figure Analysis Assignment (Faurer et al)

1. State the figure number that you are analyzing.
2. Create a simple title for your figure that describes what is being done. Example: The amount of mRNA that produces Protein X is being measured in order to determine if Treatment B alters the production of Protein X by affecting mRNA.
3. State the hypothesis that is being tested or the question being answered by this experiment. What is it that the researchers want to know by doing this experiment?
4. Each figure uses at least one experimental technique. State this technique. Find a picture to explain what this technique is and what type of information (data) is obtained by this experiment. In your explanation of this experimental technique, consider the following questions:
5. What is being measured and how?
6. What is being compared? What are the control(s) and the experimental variable(s)?
7. When looking at the data presented in the figure, answer the following question.
   1. What are the conclusions that can be made?
8. What is another experimental technique that the authors could have employed to make the same conclusion?
